# An evaluation of the effects of perioperatively administered fluids on ischemia/reperfusion injury

**DOI:** 10.12669/pjms.316.7630

**Published:** 2015

**Authors:** S. Ozciftci, M. Gamli, D. Ornek, E. Horasanli, B. Dikmen, O. Canpolat, M.Z. Ciraci, O. Kilci

**Affiliations:** 1 S. Ozciftci, Ankara Numune Training & Research Hospital, Anesthesiology & Reanimation Department, Ankara, Turkey; 2 M. Gamli, Ankara Numune Training & Research Hospital, Anesthesiology & Reanimation Department, Ankara, Turkey; 3 D. Ornek, Ankara Numune Training & Research Hospital, Anesthesiology & Reanimation Department, Ankara, Turkey; 4 E. Horasanli, Ankara Numune Training & Research Hospital, Anesthesiology & Reanimation Department, Ankara, Turkey; 5 B. Dikmen, Ankara Numune Training & Research Hospital, Anesthesiology & Reanimation Department, Ankara, Turkey; 6 O. Canpolat, Gazi University, Biochemistry Department, Ankara, Turkey; 7 M.Z. Ciraci, Gazi University, Biochemistry Department, Ankara, Turkey; 8 O. Kilci, Ankara Numune Training & Research Hospital, Anesthesiology & Reanimation Department, Ankara, Turkey

**Keywords:** Ischemia/Reperfusion injury, Intravenos fluids, Malondialdehyde, Xanthine oxidase

## Abstract

**Objective:**

To investigate the effects of normal saline (0.9% NaCl) and 6% Hydroxyethyl Starch 130/0.4(HES) solution on Ischemia/Reperfusion (I/R) injury in patients undergoing knee arthroscopy operations with spinal anesthesia using a tourniquet.

**Methods:**

The study comprised 48 ASA I-II patients undergoing knee arthroscopy with spinal anesthesia using a tourniquet. The patients were randomised into two groups and after standard monitoring two venous lines were introduced to obtain blood samples and to give intravenous therapy. In the control group (Group A) (n=21) 0.9% NaCl, 10 ml/kg/hours and in the study group (Group B) (n=19) 6% Hydroxyethyl Starch 130/0.4, 10 ml/kg/hours infusion were administered. Spinal anesthesia was applied with 12.5 mg hyperbaric bupivacaine to all patients. The tourniquet was applied and the operation was started when the sensorial block level reached T10 dermatome. Blood xanthine oxidase (XO) and malondialdehyde (MDA) levels as an indicator of ischemia and reperfusion injury were measured in samples before fluid infusion (t1), before tourniquet application (t2), 1 minute before tourniquet release (t3), and at 5 (t4) and 15 (t5) minutes after tourniquet release.

**Results:**

No difference was observed between the two groups in respect of demographic parameters, the highest block level, duration before tourniquet application and tourniquet duration (p>0.05). The MDA level after tourniquet application and 15 minutes after tourniquet release was lower in Group B (p<0.05). XO levels were not different (p>0.05).

**Conclusion:**

In this study 6% Hydroxyethyl Starch 130/0.4 solution reduced MDA level which is an indicator of lipid peroxidation. 6% Hydroxyethyl Starch 130/0.4 solution may be beneficial for Ischemia/reperfusion injuries.

## INTRODUCTION

Knee arthroscopy is a frequently performed orthopaedic operation for diagnosis and treatment. A blood-free area makes the surgical procedures considerably easier. Therefore, pneumatic tourniquets are used. Generally, the tourniquet is inflated at a systolic pressure of approximately>100mmHg. A long duration of tourniquet use (>2 hrs) may cause temporary muscle dysfunction, permanent nerve damage and ischaemia or rhabdomyolysis in the muscle tissue.^1^ When tissue is exposed to ischaemia, cellular function in the basal metabolism is impaired and a series of chemical events occur progressing as far as necrosis. The phenomenon of reperfusion, by removing the agent causing the ischaemia, attempts to re-regulate the blood flow to the tissue thus providing the energy required by the ischaemic tissue and removing toxic metabolites. However, paradoxically this causes a series of metabolic effects leading to more damage in the tissues.

Following these processes, leukocyte activation, endothelial dysfunction and tissue oedema occurs in the tissue which is reperfused with the expression of reactive oxygen metabolites, endothelin, cytokines and vasoactive mediator as reperfusion mediators.^1^-^6^ Therefore, current studies on the prevention of ischaemia/reperfusion (IR) remain important. Some of these studies have reported positive effects of the process related to fluid infusions. For example, it has been reported that microvascular reperfusion insufficiency in the skeletal muscle has been reduced by prophylactic isovolemic haemodilution,^6^ or that 6% HES 130/0.4 has positive effects on ischaemia/reperfusion injury, microcirculation, immunity, tissue oxygenation and haemodynamics.^7^-^15^ This study differs from those previous studies, as evaluation was made of XO and MDA values for ischaemia/reperfusion damage with the use of saline and 6% HES 130/0.4 solution.

Spinal anaesthesia is a frequently used anaesthesia technique in knee arthroscopy operations in which fluid replacement treatments have become important. Intravenous fluid administration is the treatment for fluid deficiency which has developed in patients for whom oral intake has been stopped because of the operation or because of hypotension which has developed associated with the degree of sympathetic blockage caused by spinal anaesthesia.^1^

Therefore, in this study it was aimed to evaluate the effects of changes in XO and MDA values on haemodynamics and ischaemia/reperfusion damage of the use of saline and 6% HES 130/0.4 solution administered perioperatively in knee arthroscopy operations under spinal anaesthesia with a tourniquet.

## METHODS

Approval for the study was granted by the Ethics Committee of the Scientific Research Evaluation Commission of Ankara Numune Training and Research Hospital (03/11/2010 decision no 20110-028). Informed consent was obtained from all the patients. The study included 48 ASA I and II status patients who underwent knee arthroscopy under spinal anaesthesia with a tourniquet. In the calculation of sample size using MDA and XO levels as the primary endpoint, it was determined that 17 patients would be required in each group for an 80% probability of detecting a difference of 0.7 mmol L in MDA values at a 5% level of significance. Preoperative haematological and chemical laboratory evaluation tests were within normal limits. Of the 48 patients, 8 were excluded from the evaluation due to haemolysis in the blood samples taken. Patients using anti-oxidant preparates such as Vitamin B and C and allopurinol, those who smoked and those with renal and metabolic diseases were excluded from the study.

All patients were evaluated one day preoperatively and were informed about the procedures to be applied and the purpose of the study. Patients were admitted to the operating room without premedication and electrocardiography (ECG), non-invasive blood pressure monitor (NIBP) and peripheral oxygen saturation (SpO2) (ADU S/5, Filland) were applied. The patients were randomly allocated into 2 groups. An IV vein was opened with an 18-gauge cannula. All patients were administered with 0.03mg/kg midazolam IV for sedation. On the contralateral arm, a 16-gauge cannula was placed to take antecubital blood samples. Infusions were started of 0.9% NaCl in the control group (Group A, n=21) and 6% HES 130/0.4 in the study group (Group B, n=19) at the rate of 10ml/kg/hour.

Heart rate, blood pressure and SpO2 values were observed at 5 minute intervals from the first values throughout the anaesthesia and operation. Heart rate, mean blood pressure, and SpO2 values were recorded as basal values, before spinal anaesthesia, immediately before tourniquet application, after tourniquet application, immediately before tourniquet release and at 1, 5 and 15 minutes after tourniquet release

All the patients were positioned laterally with the operative side in the dependent position. Following the necessary skin preparation, spinal anaesthesia was applied with a 25-gauge spinal needle at L3-4 interspinous gap (Exelint, 25 G, Los Angeles, USA). Local anaesthesia was applied to all patients as 12.5mg 5% hyperbaric bupivacaine (Marcaine Heavy, Astra Zeneca, Istanbul, Turkey) and the patients were then placed in a supine position. Throughout the operation, oxygen was administered to all patients via a nasal cannula at the rate of 2L/minutes.

When the sensorial block reached the T10 dermatome level, the tourniquet was applied and the operation was permitted to start. The time to tourniquet application and the duration of tourniquet use were recorded.

Blood samples of 4-8 ml were taken from the patients before starting the fluid infusion (T1), before applying the tourniquet (T2), at one minurte before tourniquet release (T3), at 5 minutes after tourniquet release (T4) and at 15 minutes after tourniquet release (T5). The obtained blood samples were centrifuged for 5 mins at 3000/minutes. Plasma was separated and stored at -80°C. The ratio of xanthine oxidase from the plasma samples was calculated according to the method described by Hashimoto S.^16^

The malondialdehyde (MDA) levels were measured according to the Yoshioka et al. method.^17^ A mixture of 0.5ml plasma, 2.5l trichloro-ascetic acid (200gr/L) and 1ml thiobarbituric acid (6.7 gr/L) was boiled for 30 mins. After the addition of butanol to the tube, the coloured phase was extracted by centrifugation at 3000 rpm. The butanol absorption phase was defined spectrophotometrically in a 532nm wave. Thiobarbituric acid reaction products were accepted as MDA micromoles in the plasma (nnmol/L).

Statistical analysis was performed using SPSS 15.0 for Windows software. Physical status and gender distribution were compared between the groups with the Chi-square test and the highest block level with Fisher’s Exact test. Normal distribution between the groups was defined with the Shapiro Wilk test and variance homogeneity with the Levene test. Age, weight, height, time to tourniquet application and tourniquet duration were evaluated with the Student t-test. In the inter-group and intra-group comparisons of heart rate, mean blood pressure, MDA and XO levels, the t- test was applied in dependent and independent groups. In the determination of differences within the groups and between the groups, Bonferroni correction was applied. A value of p<0.05 was accepted as statistically significant.

## RESULTS

Of the total 48 patients included in the study, 4 from each group were excluded from the evaluation due to haemolysis in the blood samples. No statistically significant difference was determined between the groups in terms of age, gender, height, weight, ASA physical status, the highest block level, time to tourniquet (Table-I).

**Table-I:** Demographic data and findings of the patients.

	Group A(n=21)	Group B (n=19)	p
ASA I	11(52.4%)	12(63.2%)	0.491
II	10(47.6%)	7(36.8%)	
Male	9(42.9%)	4(21.1%)	0.141
Female	12(57.1%)	15(78.9%)	
Highest block T10	17(81%)	18(94.7%)	0.345
Level T11	4(19%)	1(5.3%)	
Age (years)	45.52±11.129	45.84±9.221	0.923
Weight (kg)	74.238±18.941	75.157±12.010	0.857
Height (cm)	165.714±9.122	160.7895±9.354	0.100
Time to tourniquet application (mins)	40.857±17.743	46.421±15.239	0.297

A statistically significant difference was found between the groups in respect of heart beat values before and after tourniquet release. In the intragroup evaluations, in Group A, a significant reduction was found in the periods before tourniquet release and after tourniquet release compared to the basal values (Table-II).

**Table-II:** Heart beat rates of the groups at measured times.

Heart Beat Rates (beats/min)	Group A (n=21)		Group B (n=19)		p
	mean	sd	mean	sd	
t1 Basal	81.095	12.462	80.632	11.270	0.903
t 2 before application of spinal anaesthesia	79.238	11.436	85.526	11.486	0.091
t 3 before application of tourniquet	78.095	10.954	82.579	12.231	0.229
t 4 after application of tourniquet	75.286	11.283	80.737	12.569	0.156
t 5 before tourniquet release	71.048	8.464	78.263	10.759	0.023
t 61 min after tourniquet release	71.571*	9.912	79.368	10.884	0.023
t 7 5 mins after tourniquet release	70.000*	9.006	79.632	9.610	0.002
t 8 15 mins after tourniquet release	69.048*	9.064	83.789	21.872	0.007

(* = heart beat rates of Group A were found to be reduced in the periods before and after tourniquet release compared to the basal values)

In the comparison between the groups, there was no statistically significant difference in respect of mean blood pressure values apart from the basal values (Table-III).

**Table-III:** Mean arterial blood pressure values of the groups at measured times.

Mean blood pressure (mmHg)	Group A (n=21)		Group B (n=19)		p
	mean	sd	mean	sd	
t1 Basal	104.762	13.126	95.263	15.961	0.046
t 2 before application of spinal anaesthesia	102.524	12.396	98.105	17.101	0.352
t 3 before application of tourniquet	97.238	11.995	94.211	10.917	0.411
t 4 after application of tourniquet	94.286	13.331	92.421	13.120	0.659
t 5 before tourniquet release	94.000	12.787	92.000	10.333	0.592
t 61 min after tourniquet release	93.667	12.808	90.579	9.974	0.404
t 7 5 mins after tourniquet release	93.619	12.792	90.000	9.416	0.319
t 8 15 mins after tourniquet release	92.381	15.308	89.895	9.427	0.545

Comparison between the groups showed that the MDA value of Group B was found to be statistically significantly low in the periods after tourniquet application and at 15 mins after tourniquet release. In the intragroup evaluation, the MDA value was found to be statistically significantly low after tourniquet application, 5 minutes after tourniquet release and at 15 minutes after tourniquet release compared to the value before starting fluid infusion (Table-IV). No statistically significant difference was determined in the intergroup or intragroup evaluations in respect of the XO levels (Table-V).

**Table-IV:** MDA values of the groups at measured times.

MDA (nmol/mL)	Group A (n=21)		Group B (n=19)		p
	mean	sd	mean	sd	
t 1 before starting fluid infusion	4.273	1.177	3.905	1.217	0.338
t 2 before application of tourniquet	4.153	1.061	3.714	1.236	0.234
t 3 after tourniquet application	4.105	0.984	3.256*	0.802	0.005
t 45 mins after tourniquet release	3.898	1.181	3.367	1.434	0.208
t 5 15 mins after tourniquet release	3.997	1.025	3.125*	0.750	0.004

(* = MDA values of Group B were statistically significantly low after application of tourniquet and at 15 mins after tourniquet release)

**Table-V:** Xanthine oxidase values of the groups at measured times.

Xanthine oxidase (mU/L)	Group A (n=21)		Group B (n=19)		p
	mean	sd	mean	sd	
t 1 before starting fluid infusion	9.381	11.651	9.421	6.866	0.990
t 2 before application of tourniquet	8.857	10.006	12.158	9.850	0.301
t 3 after tourniquet application	7.286	7.288	11.789	9.813	0.105
t 45 mins after tourniquet release	13.238	18.311	15.105	23.723	0.781
t 5 15 mins after tourniquet release	9.571	14.260	7.421	7.328	0.559

## DISCUSSION

The effects of the perioperative use of saline and 6% HES 130/0.4 solution were examined on ischaemia/reperfusion (IR) injury in knee arthroscopy operations performed under spinal anaesthesia with a tourniquet. It was observed that in patients where 6% HES 130/0.4 was used in the perioperative period, haemodynamics were observed to be stable, malondialdehyde (MDA) levels were lower and there was no change in xanthine oxidase (XO) levels.

HES is used as a plasma substitute to correct perioperative hypovolemia. HES preparations are defined by concentration, molar substitution, mean molecular weight, the ratio of substitution, the solvent and the origin. The possible unwanted side effects of HES are anaphylactic reactions, alteration of hemostasis resulting in increased bleeding, kidney dysfunction, accumulation and pruritus.^2^

The use of the tourniquet in extremity surgeries creates a good human model for ischaemia/reperfusion injury. During ischaemia, xanthine dehydrogenase (XDH) changes irreversibly to xanthine oxidase (XO).^3^,^4^ A 2-3 fold increase in XO can be seen in rat skeletal muscle after 2 hours of ischaemia. In ischaemia events, hypoxanthine and xanthine cause the destruction of adenosine triphosphate (ATP). These two molecules function as a substrate for XO.^5^ With reperfusion, molecular oxygen reaches the tissue again and the xanthine oxidase created in the ischaemic period oxidises the hypoxanthine and xanthine oxide which have accumulated in the tissue, thus creating reactive oxygen metabolites (ROM).^4^,^5^,^16^-^18^

In the current study no significant change was determined either between or within the groups in terms of xanthine oxidase values. This may be due to the short duration of tourniquet use.

In reperfusion injury following ischaemia, the toxic metabolite, Malondialdehyde (MDA), is formed as a result of peroxidation of membrane lipids by ROM. MDA is a good indicator of lipid peroxidation and gives information about tissue damage induced by ischaemia/reperfusion.^19^,^20^ Therefore, MDA is often used in studies to show lipid peroxidation.

Although similar basal values were obtained for MDA in both groups, the reduction was gradual particularly in the group administered with 6% HES 130/0.4 and in the period when the tourniquet was applied, the values were determined to be lower. The results obtained in the current study are consistent with the study by Inan et al.^21^, which reported that 6% HES 130/0.4 solution reduced lipid peroxidation and a study by Catre et al.^22^ and Zhou et al.^23^ which reported results of an evident reduction in IR injury.

At the same time ROM cause adhesion and aggregation of neutrophils, impairment of the microvascular barrier and the no re-flow phenomenon with reduced microcirculatory blood flow. The creation of this phenomenon impairs tissue nutrition and can start a process that goes as far as cell lysis.^18^,^24^ Studies on this subject have recommended improvement of the fluid macro and micro parameters used in the perioperative period and increasing tissue oxygenation.^8^,^9^,^12^ Lang et al.^8^ applied fluid replacement treatment of Ringer lactate (RL) and 6% HES 130/0.4 to patients undergoing major abdominal surgery and an improvement in tissue oxygenation was determined in the HES group.

It has been suggested that the improvement in tissue oxygenation may be associated with improvement in the microcirculation.^8^ Popatov^9^ reported that the reason for a lower drop in mean arterial blood pressure in patients who were to undergo abdominal surgery to whom 1000ml 6% HES 130/0.4 solution was administered before epidural anaesthesia, was that stable haemodynamics were achieved without renal and haemostatic impairment and a reduced immune response was created.^9^ Dubin et al.^12^ determined an improvement in sublingual microcirculation from the application of fluid treatment with 6% HES 130/0.4 to septic patients in the early period. In the current study, better haemodynamic stability was achieved in the patients to whom 6% HES 130/0.4 was administered, which is consistent with the above-mentioned studies.

Experimental studies on this subject have reported results supportive of the results of the current study.^11^,^13^,^15^,^25^ Catre et al.^22^ compared rats which had been administered 6% HES 130/0.4 in hepatic ischaema/reperfusion with a group with no fluid administration and with a group administered 7.5% hypertonic saline and the ischaemic reperfusion injuries were found to be less in the group which had been administered 6% HES 130/0.4. This effect was reported to be due to HES molecules themselves playing a role in the reduction of the formation of ischaemia reperfusion injury rather than the effect of the volume of the HES solution.^22^ Zhou et al.^23^ determined a significant reduction in IR injury in rats administered with 10-20mL/kg HES for kidney ischaemia and reperfusion injury and reported that the reduction could be related to the adherence between neutrophils and the endothelium achieved with reduced ICAM-1 expression.^24^ Inan et al. showed positive effects of 6% HES 130/0.4 solution on ischaemia reperfusion injury and reported that the corrective effects of HES solution on microcirculation prevented the formation of free oxygen radicals and neutrophil sequestration in the reperfused tissue.^21^

HES can be used in acute normovolemic haemodilution, in the treatment of hypovolemia and as prophylaxis. With complex pathophysiological mechanisms, hypovolemia causes insufficient tissue perfusion and reduced tissue oxygenation. Therefore, besides providing macro haemodynamics, fluid treatment contributes to microcirculation and tissue oxygenation. HES solution is used successfully with these two aims. The high haemodilution capacity of HES is responsible for these effects. Pharmacologically, these include erythrocyte aggregation, platelet functions, plasma viscosity and the interactions between endothelial cells and blood corpuscles. Associated with the decreased blood viscosity, vascular resistance decreases. As a result, venous return increases and there is increased cardiac output. Consequently, this situation can be useful for tissue perfusion and oxygenation. The accumulation in the body of high molecule HES solution depends on the type of HES used. Therefore, molar substitution low HES solutions should be preferred.^9^-^15^

In conclusion, the 6% HES 130/0.4 solution used in this study provided haemodynamic stability, and during the period of tourniquet use, reduced the MDA level which is used as an indicator of lipid peroxidation but did not affect the xanthine oxidase level. Pre-loading with 6% HES 130/0.4 solution can be considered beneficial in respect of ischaemia reperfusion injury in arthroscopic knee interventions with tourniquet applications.
